# Appraisal of *Abelmoschus esculentus* L. Response to Aluminum and Barium Stress

**DOI:** 10.3390/plants12010179

**Published:** 2023-01-01

**Authors:** Rim Kouki, Nesrine Dridi, Vicente Vives-Peris, Aurelio Gómez-Cadenas, Isabel Caçador, Rosa María Pérez-Clemente, Noomene Sleimi

**Affiliations:** 1 RME-Laboratory of Resources, Materials and Ecosystems, Faculty of Sciences of Bizerte, University of Carthage, Bizerte 7021, Tunisia; 2 Department de Biologia, Bioquímica i Ciències Naturals, Universitat Jaume I, Campus Riu Sec, 12071 Castelló de la Plana, Spain; 3 MARE-FCUL, Centro de Ciências do Mar e do Ambiente, Faculdade de Ciências da Universidade de Lisboa, Campo Grande, 1749-016 Lisboa, Portugal

**Keywords:** okra, TME-tolerance, growth, Al/Ba-accumulation, nutrient uptake, secondary metabolites

## Abstract

Trace metal element (TME) pollution is a major threat to plants, animals and humans. Agricultural products contaminated with metals may pose health risks for people; therefore, international standards have been established by the FAO/WHO to ensure food safety as well as the possibility of crop production in contaminated soils. This study aimed to assess the accumulating potential of aluminum and barium in the roots, shoots and fruits of *Abelmoschus esculentus* L., and their effect on growth and mineral nutrition. The content of proline and some secondary metabolites was also evaluated. After treating okra plants with aluminum/barium (0, 100, 200, 400 and 600 µM) for 45 days, the results showed that Al stimulated the dry biomass production, whereas Ba negatively affected the growth and the fructification yield. The okra plants retained both elements and exhibited a preferential accumulation in the roots following the sequence: roots > shoots > fruits, which is interesting for phytostabilization purposes. Al or Ba exposure induced a decline in mineral uptake (K, Ca, Mg, Zn and Fe), especially in roots and shoots. In order to cope with the stress conditions, the okra plants enhanced their proline and total phenol amounts, offering better adaptability to stress.

## 1. Introduction

Trace metallic element (TME) contamination has become a major environmental problem all over the world. Increased industrialization, misguided population growth and urbanization expand the release of TMEs that compromise water and soil, and harm living biota by biomagnifying via the food chain [1]. In recent years, the risk of TME environmental pollution has rapidly increased and created turbulence—particularly in the sector of agriculture—by accumulating these elements in the soil and, therefore, in the plants. Additionally, these elements remain once introduced into the environment, contrary to organic molecules, and do not degrade [2].

Among the TMEs, aluminum (Al) is the most common component of mineral soil and ranks third among the most abundant elements of the Earth’s crust [3]. The decrease in soil pH to below 5 solubilizes the toxic forms of Al and has a toxic effect on most plants [4]; for example, trivalent aluminum (Al^3+^), which is the most abundant toxic form and has the most considerable impact on plant growth [5]. In general, Al represents a phytotoxic element and a major agronomic mishap impacting the growth and yield of many crops [4]. On the other hand, barium (Ba), an element that has not received much attention, is considered the fourteenth element by order of abundance in the Earth’s crust, with an estimated average abundance of about 425 mg·Kg^−1^ [6].

The extensive industrial use of Ba in many productions (plastics, ceramics, adhesives and drilling) enhances the release of Ba into the environment and, consequently, the contents of Ba in soil, air and water may be higher than the natural concentrations in many sites [7]. Barium phytotoxicity needs to be further investigated; only a few works have placed emphasis on the critical toxic concentrations of Ba [8]. Most studies on the absorption and translocation of Ba in plants are recent and only focus on assessing the risks of food chain contamination [9], or on the search of plants that have only been used as indicators of the presence of Ba in the soil [10].

The rate of metal accumulation and plant tolerance to heavy metals varies between species, with some elements found to be toxic even at low rates [11]. In response to the adverse effects of TMEs, plants have developed several metabolic, molecular and physiological processes that enable the avoidance or management of stressful factors, and protect cellular and sub-cellular systems from the toxic effects of reactive oxygen species using antioxidant enzymes (e.g., catalase, superoxide dismutase, peroxidase and ascorbate peroxidase, etc.) and low molecular weight antioxidants (such as ascorbate, proline, glutathione, α-tocopherols, carotenoids and phenols, etc.).

It is important to understand how exposure to the signals of an ever-changing environment manifests physiologically in plants, and how the plant behaves under stress conditions. Therefore, the purpose of this work was to evaluate the impact of the exposure of *Abelmoschus esculentus* L. plants (okra) to Al and Ba. Okra is considered a marginal crop in Tunisia, but its cultivation is expanding, since okra fruits represent an integral part of the Tunisian culinary heritage. Moreover, the concentrations of TMEs in agricultural soils are continuously increasing; therefore, a responsible recommendation is needed for the commercialization of vegetable products grown in polluted areas, in addition to the requirement of the identification of crops that tolerate metallic stress. In this context, this study was designed to investigate the capacity of accumulating Al and Ba in the roots, shoots and fruits of okra plants, and to examine the impact of these elements on their growth. Our objectives were accomplished by measuring the endogenous contents of K, Ca, Mg, Zn and Fe, as well as the determination of proline, total phenols and flavonoid contents.

## 2. Materials and Methods

### 2.1. Plant Material

A landrace variety of *Abelmoschus esculentus* L. (Marsaouia) was used in this work; the seeds were provided by the Baddar Company (Tunisia). *Abelmoschus esculentus* L. is a warm season crop that belongs to the *Malvaceae* family and is usually named okra, quiabo, bamia or lady’s finger. Okra is especially cultivated in Africa, Brazil and India, but it originates from Ethiopia, Sudan and the countries of North East Africa. Its leaves and fruits are suitable for eating.

### 2.2. Experimental Setup

The experiment was performed in the greenhouse of the Faculty of Sciences of Bizerte, under a natural photoperiod, mean temperatures (night/day) of 12/25 °C, and relative humidity between 60 and 90%. The plants were cultivated on an inert substrate (1:2 (*v/v*) a mix of gravel and perlite) and regularly irrigated three times a week with 150 mL of “Hewitt nutritive solution” [12] with a pH of 7.3. After 30 days of sowing, different doses of Al_2_O_3_ and BaCl_2_ were added to the “Hewitt solution”, and irrigations were carried out 3 times a week with 150 mL of the treatment solution. The pH treatment solutions were, on average, 5.6 and 6.6, respectively, for Al and Ba. The plants were divided into two groups: each group was divided into five groups of 10 plants; 0 (control), 100, 200, 400 and 600 μM. On the harvest day (after 45 days of treatment), the plants of each treatment were separated into roots, shoots and fruits, and then washed with cold distilled water. The roots were immersed for 10 min in cold CaCl_2_ solution (10 mM) using an aquarium pump [13] to eliminate the adsorbed trace elements. The obtained samples were divided into two groups according to the analyses to be carried out; the samples of the first group were dried in an oven at 70 °C for 10 days, and for the other group, the samples were frozen in liquid nitrogen, then kept at −80 °C, and finally, conditioned according to the analysis to be conducted.

Fresh weight (FW) and dry weight (DW) were determined before and after the drying process. The water content (WC) was determined as in Equation (1) and expressed in mL of H_2_O·g^−1^ DW:

(1) $$\mathrm{WC}=\frac{\left(\mathrm{FW}-\mathrm{DW}\right)}{\mathrm{DW}},$$

The ratio of shoot/root dry biomass (S/R), and the TME tolerance index percentage (TI %) [14] were calculated as follows (2) and (3):

(2) $$S/R=\frac{{\mathrm{DW}}_{\mathrm{Shoots}}}{{\mathrm{DW}}_{\mathrm{Roots}}},$$

(3) $$\mathrm{TI}(\%)=\frac{{\mathrm{DW}}_{\mathrm{treated}\;\mathrm{plant}}}{{\mathrm{DW}}_{\mathrm{control}\;\mathrm{plants}}}\;\times \;100,$$

All the obtained ripe fruits were collected and counted for each treatment. The fructification yield percentage was calculated as follows:

(4) $$F(\%)=\frac{\mathrm{number}\;\mathrm{of}\;\mathrm{fruits}\;\mathrm{in}\;\mathrm{each}\;\mathrm{treatment}}{\mathrm{number}\;\mathrm{of}\;\mathrm{plants}\;\mathrm{in}\;\mathrm{each}\;\mathrm{treatment}\;}\;\times \;100,$$

### 2.3. TMEs and Minerals Analysis

The measurement of the contents of TMEs and mineral elements was performed as described in the study of Sleimi et al. [15]; briefly, mineralization was conducted in Teflon bombs for 2 h at 110 °C. An amount of 45 mg of fresh plant material was extracted in a mix of acids (HNO_3_/H_2_SO_4_/HClO_4_; at the rate 10:1:0.5; *v/v/v*). The obtained extracts were diluted in 0.5% nitric acid, and finally filtered to measure the Al, Ba, K, Ca, Mg, Zn and Fe contents in the plant tissues using atomic absorption spectrometry (Perkin Elmer PinAAcle 900T, Waltham, MA, USA).

### 2.4. Translocation Factor

The translocation factor (TF) was determined by following Mattina’s Equation (5) [16]:

(5) $$\mathrm{TF}=\frac{{\mathrm{Metal}\;\mathrm{content}}_{\mathrm{shoots}}}{{\mathrm{Metal}\;\mathrm{content}}_{\mathrm{roots}}},$$

### 2.5. Proline

The proline content was analyzed as described in Bates et al. [17]. An amount of 50 mg of fresh plant material was homogenized in 5 mL of sulfosalicylic acid (3%) using a homogenizer (Ultra-Turrax, Ika-Werke, Staufen, Germany). Then, the extract was centrifuged for 20 min at 2787 g using a centrifuge (Ortoalresa, series Digicen 21, Madrid, Spain). The supernatant obtained from each sample was mixed with the ninhydrin reagent and glacial acetic acid in an 1:1:1 (*v/v/v*) proportion. The mixture was incubated in a water bath at 100 °C for 1 h and centrifuged at 484 g for 10 min. Absorbance was measured at 520 nm. The quantification was achieved through the interpolation in a standard curve prepared with commercial proline.

### 2.6. Determination of Phenolic Compounds and Flavonoids

To determine the amounts of phenolic compounds, 30 mg of the dried plant matter was mixed with 10 mL of methanol 80% and incubated in the dark overnight. The extract was centrifuged for 30 min at 629 g using a centrifuge (MPW-351 R, GmbH & Co. KG, Bremen, Germany). After filtration, the supernatants were used to spectrophotometrically estimate the total phenol contents at 765 nm, as described in the study of Velioglu et al. [18] and slightly modified by Bouslimi et al. [19]. The flavonoid contents were measured at 430 nm as previously described by Quittier et al. [20].

### 2.7. Statistical Analysis

All the samples were analyzed for at least five replicates; the average values and standard deviation (±) are displayed in vertical bars in the figures. The impacts of TME on the variability of the studied parameters were examined using a single-factor analysis of variance (ANOVA 1) by STATISTICA software to determine if a given factor had a significant effect. Concerning the comparison of the means, Tukey’s HSD test was used, which provides the significant differences of these data at *p* ≤ 0.05. An association analysis between the studied parameters was carried out by correlation circle from a principal component analysis (PCA) using STATISTICA 8.0 software.

## 3. Results

### 3.1. Growth

#### 3.1.1. Plant Morphology

**Figure 1:**
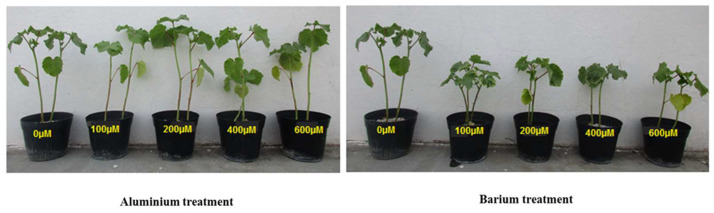
Morphology of *Abelmoschus esculentus* L. plants treated with increasing doses of Al (**left** picture) and Ba (**right** picture).

**Figure 2:**
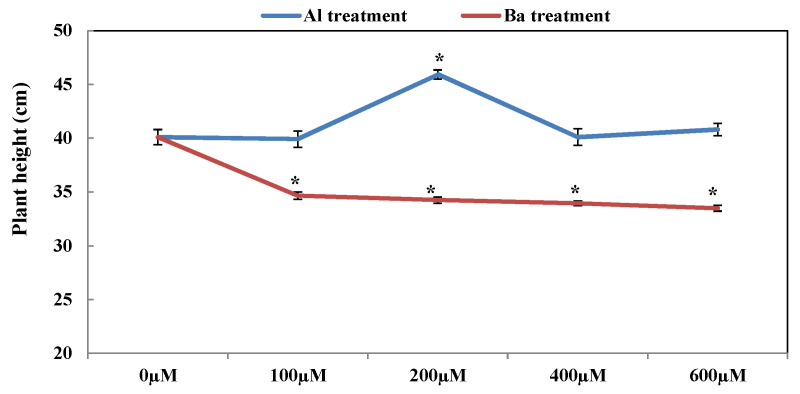
Variation of plant height in *Abelmoschus esculentus* L. plants treated with increasing doses of Al and Ba. Data are presented in mean values ± SD, *n* = 10. (*) significantly different at *p* ≤ 0.05.

After 45 days of the exposure of *A. esculentus* L. plants to two different TMEs (Al or Ba), the plants of each treatment presented some morphological differences compared to the untreated plants (Figure 1). The plants that were treated with Al were capable of growing and developing normally under stress conditions; our observations revealed that okra did not show any visible signs of Al toxicity (neither foliar chlorosis nor necrosis). Furthermore, the plants that were treated with 200 µM of Al showed a significant enhancement of 14.53% in height (Figure 2). On the other hand, the plants that were exposed to Ba-induced stress showed a visible significant reduction in height with all Ba doses as compared to the control plants. Maximum reduction (16.48%) was observed in the plants that were treated by 600 µM of Ba (Figure 2). The results also showed that Ba induced foliar yellowing in some leaves, especially at high doses (Figure 1).

#### 3.1.2. Dry Biomass Production

**Figure 3:**
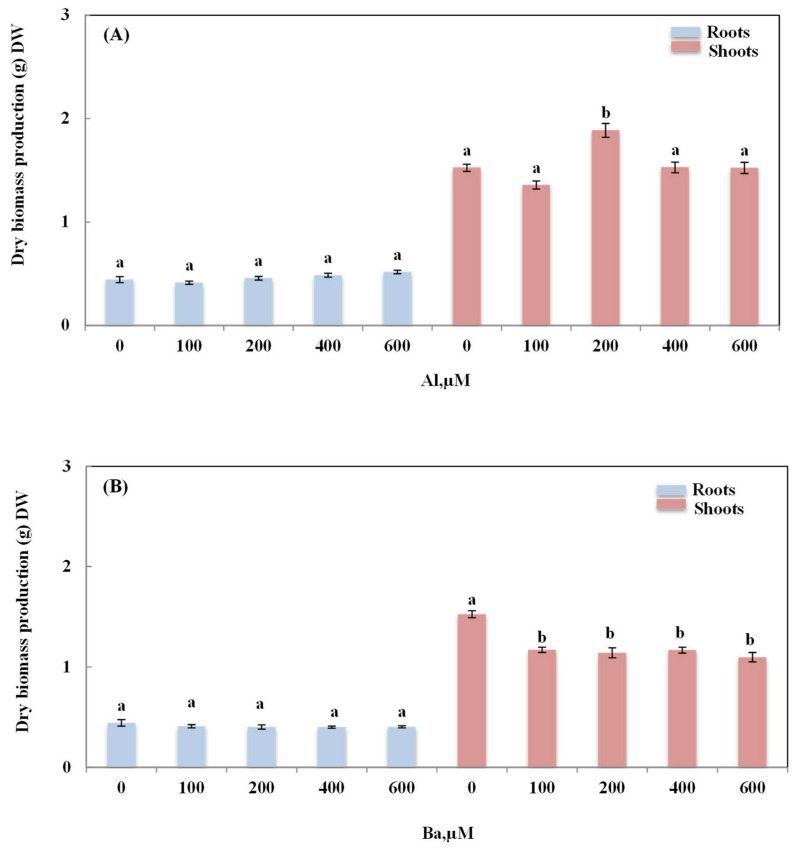
Variation of dry biomass production of roots and shoots of *Abelmoschus esculentus* L. plants treated with increasing doses of Al (**A**) and Ba (**B**). Data are presented in mean values ± SD, *n* = 10. Bars marked with different letters represent statistical differences at *p* ≤ 0.05.

The evaluation of the effects of Al and Ba on the growth of *A. esculents* L. was also based on the assessment of the dry biomass production; the results are presented in Figure 3 and Table 1. Data revealed that both elements had opposite effects on the growth of the okra plants. In the Al treatment, besides the notable increase in plant height in plants watered with 200 µM of Al, a stimulatory effect was also noticed in the dry biomass of the shoots. Dry biomass production significantly increased by 23.73% (*p* ≤ 0.05) as compared to the control, and this was also confirmed by the increase in the shoot/root ratio (up to 16.49%), mainly in plants treated with 200 µM of Al (*p* ≤ 0.05, Table 1). In roots, Al did not induce any significant variation in the dry biomass production, whereas Ba treatment caused a significant decrease in the dry biomass production of the shoots with all the doses used in the treatment (the maximum decrease reached 28.01%, which was observed in plants grown in presence of Ba 600 µM). Therefore, a significant decrease of 24.77% was also recognized in the shoot/root ratio (*p* ≤ 0.05; Table 1).

#### 3.1.3. Tolerance Index

The study of the variation of the tolerance index percentage (TI %) (Table 1) in okra plants treated with Al or Ba reminded us of the variation of dry biomass production; a noteworthy improvement of 27.67% was observed in the shoots of plants treated with 200 µM of Al. In the roots, the TI was stimulated in plants watered with 400 and 600 µM of Al, reaching values of 112.75 and 121.8%, respectively. On the other hand, Ba treatment caused a decline in TI % in the aboveground parts of the plants treated with all the used doses, and a maximum decrease of 32.75% was observed with 600 µM of Ba. Similarly, okra roots showed a decrease in TI % values, reaching 12.52% in plants subjected to 400 µM of Ba.

#### 3.1.4. Water Content

According to our findings in Table 1, the Al and Ba treatments induced water status alterations in the okra plants. In the shoots, water content (WC) was enhanced as compared to the control, especially in the presence of 600 µM of Ba, where the values significantly increased by 41.44% (*p* ≤ 0.05). In the roots, Al caused an inhibition of WC as compared to the control plants, especially with 600 µM. However, Ba increased the moisture content in the roots of plants treated with doses above 200 µM (*p* ≤ 0.05).

#### 3.1.5. Fructification Yield

The results presented in Table 2 illustrate the effect of the exposure of okra plants to Al and Ba on the fructification yield percentage. For Al, as compared with the control plants, the yield was not highly affected, except for the reduction of 20% and 10% that was observed in plants subjected to 100 and 600 µM Al, respectively. Regardless of this decrease, okra presented a normal fructification yield as compared to the controls. On the other hand, and similarly to the dry biomass production findings, Ba negatively affected the fructification of the studied plant with all the used doses.

### 3.2. TME and Minerals Accumulation

#### 3.2.1. Al and Ba Accumulation

**Figure 4:**
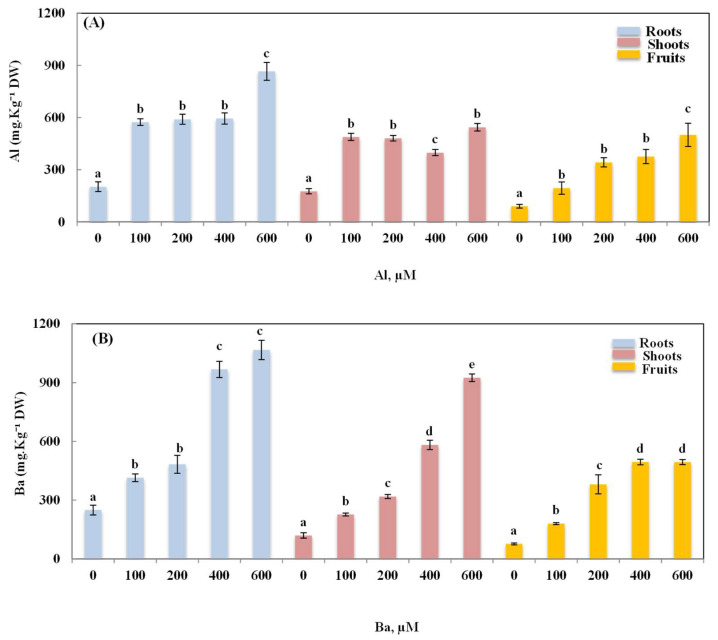
Variation of Al contents (**A**) and Ba contents (**B**) in the roots, shoots and fruits of *Abelmoschus esculentus* L. plants subjected to increasing doses of Al and Ba. Data are presented in mean values ± SD, *n* = 10. Bars marked with different letters represent statistical differences at *p* ≤ 0.05.

According to the obtained results, Al exhibited a distribution among the different parts of the studied plant (Figure 4). The results showed a significant increase in Al content in all the organs of the plants treated with Al, regardless of the concentration. The highest accumulated contents of Al were observed in the plants treated with 600 µM of Al. Al was mostly retained in the underground parts of the plant, where the accumulated contents were around three times higher than the roots of the control plants, with an increase of 326.35%. In shoots and fruits, the accumulation of Al showed an increase of 208.40 and 568.21%, respectively.

The same observations were noted in plants treated with Ba (Figure 4). The endogenous contents of this element significantly increased (*p* ≤ 0.05) along with the increase in the Ba doses used in the treatment. Ba was also accumulated in all parts of okra plant, but mainly in the roots. The maximum increase in the accumulated contents of Ba reached 327.8 and 674.9%, in the roots and shoots of plants treated with 600 µM of Ba, respectively. In the fruits, the maximum increase in the recorded contents of Ba reached 458.58 and 458.08% with 400 and 600 µM, respectively.

#### 3.2.2. Translocation Factor of Al and Ba

Examining the translocation of Al from the roots to the shoots (Table 3), we noticed that the translocation factor (TF) values were higher in plants subjected to 100 and 200 µM concentrations and tended to decline slightly with increasing the concentrations of Al in the irrigation solution. The TF values were found to be lower than 1 (TF < 1) with all the applied doses of Al. On the other hand, data showed an increase in TF values along with the increase in Ba doses, although the TF values were also lower than 1, especially in plants irrigated with doses higher than 200 µM of Ba.

### 3.3. Mineral Accumulation

The exposure to Al and Ba stress resulted in a perturbation in the uptake of minerals by okra plants. Table 4 presents the variations of potassium (K), calcium (Ca), magnesium (Mg), zinc (Zn) and iron (Fe) uptake in the roots, shoots and fruits under the effect of increasing Al and Ba doses.

#### 3.3.1. Potassium

Our results revealed that Al and Ba exposure caused a significant reduction in K^+^ levels with all the doses used in the treatment (*p* ≤ 0.05). The inhibitory effect was more noticeable in the Al treatment, where the maximum reduction was 36.44% in the roots and 31.09% in the shoots with 600 µM, while for Ba it was about 24.98 and 27.67% in roots and shoots, respectively. Fruits did not present any significant variation in K content under both treatments, despite the slight increases observed with all the doses of TMEs (Table 4).

#### 3.3.2. Calcium

The results also showed that the Ca contents were negatively and significantly affected by Al and Ba stresses. For Al, this significant reduction was mainly observed with 200, 400 and 600 µM of Al; the maximum reduction was 22.23% in the roots of plants watered with 400 µM of Al and 22.27% in the shoots with 600 µM of Al (*p* ≤ 0.05). Concerning the plants treated with Ba, the decrease was concomitant with the increase in Ba doses; in the roots, a decrease of 19.59% was noted with 200 µM of Ba, and in the shoots of plants treated with 400 µM of Ba, the Ca contents also decreased, by 18.2% (*p* ≤ 0.05). Moreover, only the fruits obtained from Ba-treated plants showed a significant decline in Ca content (28.67%) compared to the control (*p* ≤ 0.05).

#### 3.3.3. Magnesium

Compared to the other minerals tested in this study (Table 4), the decrease in the Mg contents in okra plants was lower in the Al treatments. The roots of the plants treated with 200 and 400 µM of Al showed a significant decrease in Mg content of 17.90 and 19.81%, respectively (*p* ≤ 0.05). In the aboveground parts of the plant, the Mg contents significantly decreased to 22.01% with 600 µM of Al. However, the fruits did not show any significant variation in Mg content in the Al-treated plants. The Mg content was not altered in the Ba-treated plants regardless of the Ba concentration and the considered organ (Table 4).

#### 3.3.4. Zinc

Okra plants exposed to TME stress showed a sharp decrease in Zn content with all the used doses in both treatments (Al or Ba; Table 4). The roots and shoots of plants treated with 400 µM of Al presented a maximum decrease in Zn contents of 42.81% and 66.01%, respectively. A similar effect was also noticed in plants treated with Ba; Zn absorption was significantly inhibited in okra as compared to the control, with the maximum reduction reached up to 45.96%, 75.86% and 13.75% in the roots, the shoot and the fruits of plants treated with 200 µM of Ba, respectively (*p* ≤ 0.05).

#### 3.3.5. Iron

The results revealed that okra plants experienced a decline in Fe uptake under Al and Ba stresses (Table 4). The Fe contents were inhibited in plants exposed to Al doses higher than 200 µM. In the roots, Fe endogenous content was significantly reduced to 36.58% and 48.12% with 200 and 400 µM of Al, respectively (*p* ≤ 0.05), whereas in the fruits, the highest decrease was noticed at 600 µM of Al reaching 28.08% compared to the control (*p* ≤ 0.05). Likewise, Ba stress sharply inhibited the uptake of Fe by okra plants; the Fe contents were reduced by up to 92.23% and 89.35% in the roots and shoots with 600 µM, respectively. Concerning the fruits, the maximum decrease reached up to 63.41% in 400 µM of Ba (*p* ≤ 0.05).

### 3.4. Proline

After 45 days of Al treatment, there was a considerable variability in the proline contents in okra plants. Compared to the control, the proline content significantly increased in the roots, shoots and fruits, where maximum stimulation reached 22.62%, 59.81% and 29.27% in the highest Al dose; 600 µM, respectively (*p* ≤ 0.05). Likewise, in the roots, high doses of Ba significantly stimulated the proline contents to reach 28.24% and 28.73%, with 400 and 600 µM, respectively, as compared to the control plants (*p* ≤ 0.05). Moreover, in the aerial parts, Ba stress generated a significant increase in the proline contents, with a maximum stimulation in the shoots and fruits of 54.63 and 28.67%, respectively, in the presence of 200 µM of Ba (*p* ≤ 0.05), compared to the control plants (Table 5).

### 3.5. Secondary Metabolites

#### 3.5.1. Total Phenols

The exposure of okra plants to Al and Ba stress induced an increase in total phenols (TP), especially with the highest TME doses (Table 5). This increase was more obvious in the shoots than in the roots. The results showed that 600 µM of Al significantly stimulated the TP rates to reach 40.2% in the roots and 147.21% in the shoots (*p* ≤ 0.05). Likewise, for Ba treatment, the maximum increase was 35.45% in the roots and 60.85% in the shoots (*p* ≤ 0.05). However, the fruit TP content did not present any significant variation in both treatments (*p* ≤ 0.05).

#### 3.5.2. Flavonoids

The data presented in Table 5 show that the flavonoid contents were negatively affected by TME stress, as compared to the untreated plants. The okra plants experienced a significant decline in the concentration of these compounds in the roots and shoots, especially at high TME doses (*p* ≤ 0.05). The maximum decrease was observed with 600 µM of Al and reached 96.14% in the roots and 49.24% in the shoots. For the Ba treatment, the maximum decrease reached 88.18% in the roots of plants treated with 200 µM, and 64.24% in the shoots of those treated with 600 µM of Ba (*p* ≤ 0.05).

### 3.6. Principal Component Analyses (PCA)

A PCA analysis was carried out in order to study the impact of Al and Ba on the different studied parameters: growth, mineral nutrition, proline and secondary metabolites in okra plants, as well as the correlation between the different parameters.

**Figure 5:**
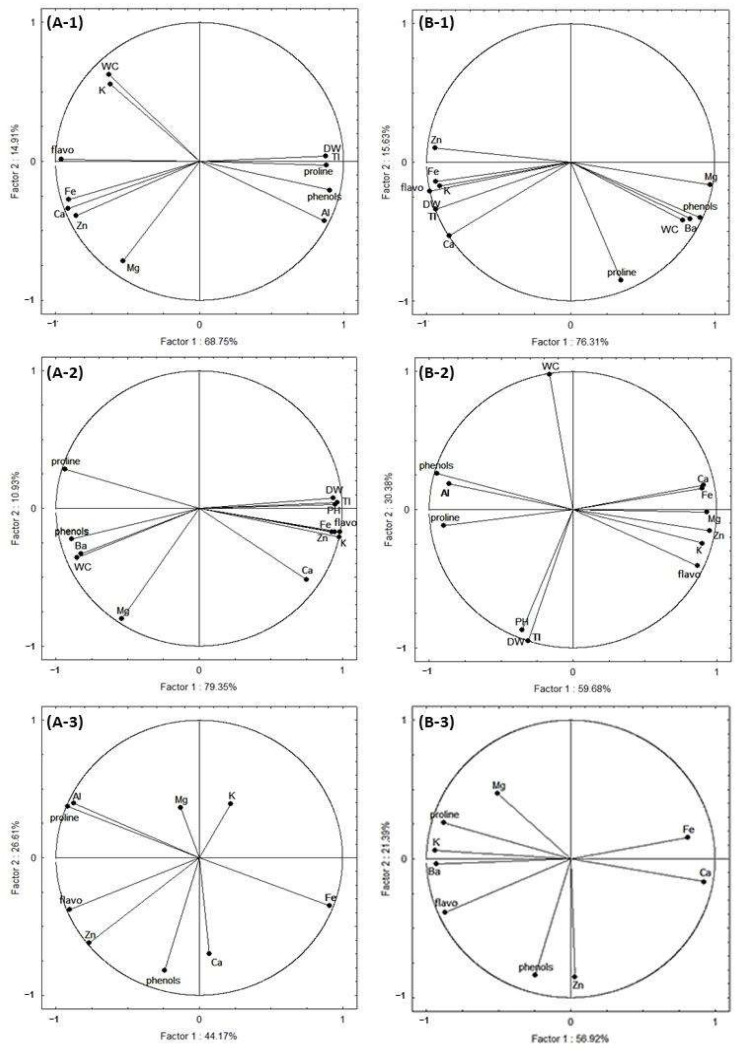
Correlation circle from the principal component analysis (PCA) of aluminum (Al) and barium (Ba) contents; dry biomass (DW); water content (WC); tolerance index (TI); plant height (PH); mineral nutrient contents; potassium (K); calcium (Ca); magnesium (Mg); proline; total phenols (phenols); flavonoids (flavo); data of the roots, shoots and fruits of *Abelmoschus esculentus* L. plants subjected to increasing doses of Al and Ba. (**A-1**,**A-2**,**A-3**) means roots, shoots and fruits of plants treated by aluminum, respectively. (**B-1**,**B-2**,**B-3**) means roots, shoots and fruits of plants treated by barium, respectively.

Figure 5 illustrates the multiple factor analysis of Al and Ba effects on the okra plants. The obtained results showed a high significance; for plants treated with Al, the total variance reached 83.66%, 90.28% and 70.78% in the roots, shoots, and fruits, respectively, while in the Ba treatment, the total variances were revealed to be even higher than in the Al treatment, representing 91.94%, 90.06% and 78.31% in the roots, shoots and fruits, respectively.

The Al content was positively correlated to DW, TI, proline and total phenols in the roots (Figure 5(A-1)) and negatively correlated to WC, most of the mineral contents (K, Ca, Zn and Fe) and flavonoids. In the shoots, proline and total phenols presented a positive correlation, but K, Zn and Mg were negatively correlated with the Al content (Figure 5(A-2)). In the fruits, there was a positive correlation between the Al content, proline and flavonoids, while the Fe content presented a negative correlation with Al (Figure 5(A-3)).

Concerning Ba treatment, it was revealed that the Ba root content (Figure 5(B-1)) was positively correlated with the Mg contents, total phenols and proline, and negatively correlated with the K, Zn and Fe contents and flavonoids, whereas the Ba contents in the shoots (Figure 5(B-2)) presented a positive correlation with WC, phenols and proline and a negative correlation with K, Fe contents, and flavonoids. It was observed that, in the fruits, the Ba content was positively correlated to the K contents, proline, and flavonoids, whereas the Ca and Zn contents presented a negative correlation (Figure 5(B-3)).

## 4. Discussion

### 4.1. Growth

As a part of investigating how Al and Ba can affect the growth of okra plants, the results revealed that these elements had opposite effects on the development and the biomass production. *A. esculentus* L. maintained good growth and tolerated exposure to Al. This was also highlighted with the increase in TI values.

In the literature, Al was found to adversely affect plant growth and metabolism. This element is implicated in the inhibition of primary root growth [21] and the attenuation of the photosynthetic performance [22,23]. For example, the exposure of cucumber plants to Al stress caused a decrease in biomass production in the roots [24]. Pirzadah et al. [25] revealed that in two buckwheat species, fresh biomass was significantly reduced to 58.15% and 58.09%, respectively, in *F. kashmirianum* and *F. tataricum* with 300 μM of Al.

Further, Al-accumulator plants are able to tolerate elevated concentrations of Al without suffering from phytotoxicity. It was reported that *Camellia sinensis* (L.) plants were able to tolerate up to 3200 µM of Al through an increase in the capacity of the scavenging and detoxification of ROS [26]. In *Camellia japonica,* 500 and 1000 µM of Al promoted growth by enhancing the levels of photosynthesis, increasing the contents of soluble sugar and total soluble protein [27]. Otherwise, the stimulatory effect of Al on plant growth has been frequently noticed, although it is regarded as a non-essential nutrient. The increase in growth under the effect of Al has been observed in several plant species such as corn, where 48 µM of Al^3+^ promoted leaf growth [28], as well as in *Quercus serrata*, where this element enhanced growth and photosynthetic activity [29]. The beneficial influences of Al on plants have also been explained by its role in enhancing phosphorus (P) availability and use efficiency by plants, and alleviating H^+^, manganese and iron toxicity in acidic conditions. In addition, Al is involved in the activation of genes associated with abiotic stress tolerance, mainly those that are responsible for oxidative stress response, low P response, and organic acid secretion [30]. Moreover, the application of Al increased chaperone protein rates in the leaves of citrus plants [31]. Indeed, Al toxicity affects the refolding of proteins, leading to serious protein denaturation. Chaperone proteins are involved in reestablishing the normal protein conformation and maintaining cellular homeostasis; this is achieved by preventing the formation and aggregation of misfolded proteins in the presence of Al and ensuring that these proteins are refolded [32].

Furthermore, studying the effect of the increasing doses of Ba on the growth of okra plants revealed that this element caused a decline in biomass production, mainly in the aerial parts, along with an increase in the used doses of Ba. Generally, the inhibitory effect of Ba on growth, as well as the development alteration, are the most obvious symptoms of its phytotoxicity. Similar findings were obtained in cucumber plants subjected to Ba stress, where the dry biomass production showed a remarkable decrease [33]. Equally, the stress caused by Ba exposure inhibited the growth of the shoots in *Cakile maritima* [19]. Moreover, high levels of Ba caused phytotoxicity in Tanzania guinea grass through the alteration of various mechanisms related with nutritional status and growth, resulting in the death of basal sprouts and the weakening of adult plants [34]. Suwa et al. [9] explained the decline of growth under Ba stress by the reduction in CO_2_ assimilation caused by the reduction in photosynthetic activity.

According to the results of TI % in Table 1, okra plants presented a better performance when exposed to Al stress as compared to those treated with Ba, which indicates that okra is more sensitive to Ba stress and has a lower ability to cope with it. This was also confirmed by the difference obtained in the fructification yields. Compared to the control, the okra plants maintained normal fructification in the Al treatment; meanwhile, Ba caused a prominent reduction in the number of fruits. Similarly, in tomato, arsenic exposure reduced fruit yield [35]. Likewise, Shekar et al. [36] observed that mercury caused a reduction in flowering, and thus, fructification. Abiotic stress can affect the fructification process and fruit yield. Shrivastava and Kumar [37] claimed that salinity interferes with reproductive development through inhibiting the elongation of stamen filaments and microsporogenesis, increasing programmed cell death in certain types of tissues, ovular abortion, and the senescence of fertilized embryos.

Moreover, previous studies have shown that exposure to TME stress disturbs the water status in plants. For instance, in cucumber plants treated with Al [24] and Ba [33], the water content in the roots increased compared to the control, which agrees with our findings. Other studies revealed that in soybean, Ba treatment did not affect the water potential or relative water content [9]. However, our data showed that using high doses of Al and Ba (600 µM) induced a slight decline in water status in the roots. Some authors explained that the lack of hydration is caused by ineffective water uptake due to the participation of the aquaporins in Al transport [38].

### 4.2. TMEs Accumulation

Usually, Al is accumulated in the underground parts of the plant, and a minor portion is translocated towards the shoots in most plant species. For example, in *Cucumis sativus* plants, it was revealed that this element was mostly trapped in the roots, and low amounts were translocated to the shoots [24]. Yet, some species, such as hydrangea, tea, and buckwheat, are able to retain large contents of Al in the tissues of the aerial parts of the plants, without showing any symptoms of Al toxicity [39]. Some of these plants have proved to be well compliant with maximum metal transferred to aboveground parts, which may be an adaptation strategy to minimize the vulnerability to Al of root tissue, thereby ensuring its avoidance [40]. The contents of Al in the different parts of the okra plants increased along with the increase in the used doses in the experiment. In reality, Al-hyperaccumulators can accumulate more than 1000 µg·g^−1^ in the dried tissue of their aboveground parts [41], with the maximum accumulation of Al in the shoots of our plants reaching 545.06 mg·Kg^−1^ of DW; thus, on the authority of our findings, *A. esculentus* L. can be considered only as an Al-accumulator due to its ability to accumulate important contents of Al.

Studies of the elementary composition of plants have revealed that Ba is considered a plant-biophilic element, and it is absorbed through active transport by following the path of certain plant nutrients, or because it might play the role of a plant nutrient itself [42]. Our data showed that Ba exhibited accumulation in a dose-dependent manner and distribution in all the parts of the plant, with preferential retention in the roots. In most plant species, the average Ba content ranges from 2 to 13 mg·Kg^−1^, and the highest accumulated rates of Ba were noticed in Brazil nuts (3000–4000 mg·Kg^−1^) [43]. In cucumber plants, the Ba contents reached 6.93 mg·g^−1^ in the roots and 6.62 mg·g^−1^ in the shoots in the presence of 500 µM of Ba [33]. At present, only a few plant species have been identified as accumulators of Ba [44]. In Warsaw, Poland, great mullein plants (*V. densiflora*) were the prevalent colonizers of a wasteland near an industrial plant, and the shoots accumulate high Ba-contents equivalent to 343.6 mg·Kg^−1^ of DW; therefore, it was identified by Kowalska et al. [10] as a Ba-accumulator plant. However, the Ba levels found in the shoots of the okra plants were even higher (925 mg·Kg^−1^ of DW); thus, our plant species can be defined as a Ba-accumulator plant.

In this work, the studied plant presented high TF values (0.854 with 200 µM of Al and 0.845 with Ba at 600 µM) but always lower than 1, highlighting the ability of okra plants to accumulate high contents of Al and Ba in the roots more than in the harvestable aboveground parts, and maybe the involvement of the roots in a detoxification strategy, highlighting the possibility of using okra for phytostabilization purposes.

Since the fruit of okra is edible, it is interesting to investigate its accumulator potential of Al and Ba. In the purchased vegetables, Al could also be accumulated, for example, in carrot (0.096 mg·g^−1^), cucumber (0.356 mg·g^−1^), pumpkin (0.929 mg·g^−1^) and parsley (1.06 mg·g^−1^) [45]. The okra fruits obtained from the plants treated with 600 µM of Al (the maximum used dose) accumulated 0.501 mg·g^−1^ of DW. According to the FAO/WHO [46], the weekly Al dietary intake corresponded to 2 mg/Kg/body weight. In this case, the obtained okra fruits were still safe for human consumption, even after the exposure to Al, but in moderation. For example, a 75 Kg–person should not consume more than 3.0 Kg/week, taking into account the water content in the sample.

The contents of Ba in the vegetables sampled from Ba-polluted sites reached 1.398 mg·Kg^−1^ of DW in corn, 3.021 mg·Kg^−1^ of DW in eggplant and 2.641 mg·Kg^−1^ of DW in marrow [47]. In this study, *A. esculentus* L. presented efficiency in accumulating Ba in the fruits, with the maximum Ba accumulation reaching 494.11 mg·Kg^−1^ of DW, and according to the FAO/WHO [46], this value exceeded the WHO limit of 0.850 mg·Kg^−1^ of DW.

### 4.3. Mineral Uptake

Minerals are involved in the composition of many structural component proteins and carbohydrates; moreover, these elements can act as activators of some enzymes, and they are involved in maintaining the osmotic balance [48]. Plants exposed to TME stress may show a reduction in the uptake of some cations involved in the plant metabolism. For example, in *Atriplex halimus* treated with 400 µM of Cd, the Mg^2+^ and K^+^ contents in the leaves were negatively affected [49].

Previous studies have reported that the accumulation, acquisition, localization and utilization of most mineral elements can be altered by Al [5]. In *Camellia japonica*, 1000 µM of Al induced a decrease in the endogenous concentrations of Mg and Ca [27]. According to de Freitas et al. [50], in upland rice treated with 1110 and 1480 µM of Al, the contents of K, Ca, Fe and Zn were strongly reduced because of the competition of Al and minerals for the sites of the uptake on influx channels, and the transporters of mono- and divalent cations. In reality, the interaction of Al with plasma membrane modifies its structure [48]. Moreover, Al may decrease the negativity of the cell surface in the roots, reducing Fe uptake on the cell surface. Wang et al. [28] suggested that Al and Fe can have similar absorption mechanisms.

Despite the observed inhibition in mineral uptake under Al stress, the okra plants were not severely affected, and no morphological symptoms of mineral deficiency were recognized. In fact, amino acids, such as tryptophan, methionine and glycine, can supply plants with nutrients by acting as growth promoters [51]. Furthermore, under water deficiency, plants produce osmolytes such as proline, which can help to prevent nutrient deficiencies to alleviate the effects of this stress [52].

Our data showed that Ba also acted as a limiting factor for the assimilation of minerals in okra plants. In accordance, Ba treatment induced a decrease in K, Ca and Mg rates in the shoots of soybean plants [9]. The results reported by Llugany et al. [8] also showed an inhibition in the K and Ca contents in the shoots of bush bean plants.

Ba toxicity is likely due to the antagonistic interactions between K and Ba. In our study, potassium was the most Ba-sensitive nutrient. In reality, K and Ba have a very comparable ionic radius; despite this, K does not strongly compete for binding sites requiring divalent cations, and Ba is actually an inhibitor of the inward K^+^-channels [53]. In addition, previous observations by Wallace and Romney [54] proved that Ba may interfere with the Ca nutrition of plants, which was also observed and confirmed in our results.

Otherwise, in Ba-treated plants, the Mg contents did not show a decrease; they were even slightly increased. The same effect was observed in *Limbarda crithmoides* and *Helianthus annuus* subjected to Ba stress [55]. Rengel et al. [56] claimed that Mg can be involved in the mitigation of metallic stress in plants by reducing the negative electrical potential, and thus, metal ion activities at the plasma membrane surface, or by enhancing the vacuolar sequestration of heavy metals via increasing H^+^-pumping activity in the tonoplast.

On the other hand, Al and Ba can reduce the availability of essential nutrients in the culture medium due to ionic interactions, even if these nutrients are present in sufficient concentrations for the plants (K^+^: 4.9 mM; Ca^2+^: 3.5 mM; Mg^2+^: 1.5 mM)

### 4.4. Proline

The increase in the proline levels in plants under several types of stress has been frequently reported. For example, endogenous concentrations of proline showed an increase in *Plantago maritima* plants exposed to salt stress [57] and in *Cucurbita pepo* plants treated with increasing doses of Cd [58].

Our data support these results, proving a subsequent accumulation of proline under Al and Ba treatments. In *A. esculentus* L. plants, Al and Ba gave rise to proline amounts in the different parts of the plant. The increase in its endogenous concentrations was mainly noticed with the highest TMEs doses. This increase in proline contents was also reported in mung bean subjected to 1 mM and 10 mM of Ba [59], and in Tanzania guinea grass under 5 and 20 mM of Ba [34].

Indeed, proline is an imino acid that is considered a common metabolite in crop plants. According to Garg and Neha [60], it plays various roles in undesirable environmental conditions: (i) proline acts as a chemical chaperone by regulating the pool of glutathione (GSH) via the sustainment of the redox potential of NADPH/NADP^+^, which leads to an increase in the synthesis of phytochelatins and the formation of metal–thiolate complexes in the vacuole, thus, protecting against the metallic stress. (ii) Proline can act as a metal chelator through the formation of proline–metal complexes of proline had a protective role in maintaining the integrity of DNA.

The accumulation of proline is associated with abiotic stress tolerance, and its increased concentrations have generally been stated in tolerant species as compared to sensitive ones. Therefore, the ability of okra plants to tolerate the induced stress and survive without being significantly affected can be related to the involvement of proline in the mitigation of the harmful effects of TMEs.

### 4.5. Polyphenols and Flavonoids

The level of phenols in the plant tissue can be a good indicator for predicting the extent of tolerance to abiotic stress in plants, and which varies widely in different plant species under various external factors. Plants that exhibit an improvement of polyphenols synthesis under abiotic stresses usually show better adaptability under limiting environments [61]. For example, the phenylpropanoid biosynthetic pathway was stimulated by TMEs in plants through the up-regulation of key biosynthetic enzymes activities such as PAL, G6PDH, SKDH and CADH [62].

In conformity, the polyphenol contents were stimulated after the exposure of the okra plants to increasing doses of Al and Ba. This behavior was also observed in *Brassica juncea* and *Cackile maritima* plants subjected to Ba stress [19], as well as in *Helianthus annuus* plants treated with La and Ce [63]. Indeed, phenolic compounds play the role of antioxidants by participating in ROS scavenging, catalyzing the reactions of oxygenation through the formation of metallic complexes, and via the inhibition of the activities of oxidizing enzymes [64]. In plants, polyphenols are involved in several physiological processes to ameliorate the adaptability and tolerance of plants under unfavorable conditions [65]. Thus, the increase in polyphenol contents in the tissues of okra plant improves its efficiency in coping with the TME stress. This increase was more noticeable in the plants exposed to Al stress, which may confirm that okra has more susceptibility to these compounds, and thus, may be more tolerant than plants treated with Ba.

Flavonoids also play a crucial role in the antioxidant response of plants exposed to TMEs stress by being a part of the ROS scavenging system and enhancing the process of metal chelation, which helps to minimize the detrimental levels of hydroxyl radicals in plant cells [66]. This fact is in agreement with the previous reported observations that the metal excess increased the levels of flavonoids in plants [67]. In this work, it was revealed that the flavonoid contents decreased in the okra plants exposed to Al and Ba, which could be due to the non-intervention of flavonoids in the detoxification of oxidative stress products. This behavior may be attributed to the fact that Al and Ba could interfere with the biosynthesis pathway of flavonoids. In addition, Berni et al. [68] suggested that plants can primarily invest in the phenolic acid biosynthesis pathway (hydroxycinnamic acids) under stressful conditions and deactivate genes involved in the following steps (that lead to the formation of anthocyanins and flavonoids), in order to save energy, while being able to cope with stress via phenolic compounds.

## 5. Conclusions

Since Al and Ba are widely dispersed elements that can be found in soils, drinkable water and even food, and since human health is a priority, it is always interesting to investigate species that present an ability to grow normally in contaminated soils and produce edible fruits that respect the standards of the FAO/WHO. In summary, *Abelmoschus esculentus* L. plants exposed to two different TMEs showed a better performance when subjected to Al as compared to Ba stress; the plant growth was ameliorated with Al, whereas Ba caused an inhibition of plant biomass production and a reduction in plant height. These obtained results were confirmed by the TI values. Moreover, the okra plants presented high efficiency in accumulating Al and Ba. In fact, Al and Ba exhibited a disturbance in all the different parts of the plant following the sequence: roots > shoots > fruits. In addition, contrary to Ba, the fruits obtained from the Al treatment are suitable for human consumption, since they do not exceed FAO/WHO standards. The preferential retention of both elements in the roots offers encouraging perspectives for using okra for phytostabilization purposes in Al/Ba-polluted soils. The TMEs exposure resulted in an alteration of the mineral uptake in the okra plants; K, Ca, Mg, Zn and Fe presented a decline, especially in roots and shoots, with increasing doses of Al and Ba. Despite the negative effects of TME, the okra plants presented an ability to survive in an environment contaminated with these elements; this may be linked to the development of some defense mechanisms against the induced stress, such as the increase in proline rates and secondary metabolites.

## Figures and Tables

**Table 1:** Variation of the shoot/root dry biomass ratio (S/R), tolerance index (TI) and water content (WC) in *Abelmoschus esculentus* L. plants subjected to increasing doses of Al and Ba (0, 100, 200, 400 and 600 µM). Values are mean ± SD (*n* = 10); (*) significantly different at *p* ≤ 0.05.

Al (µM)	S/R	TI %			WC (mL·g^−1^ DW)	
		Roots	Shoots	Entire Plant	Roots	Shoots
0	3.63 ± 0.2				13.85 ± 0.24	7.31 ± 0.14
100	3.31 ± 0.1	91.32 ± 4.44	88.26 ± 2.63	88.06 ± 3.17	13.44 ± 0.27	8.38 ± 0.23
200	4.23 ± 0.27 *	103.98 ± 5.06	127.67 ± 5.09	122.8 ± 4.14	13.08 ± 0.56	6.74 ± 0.13
400	3.23 ± 0.17	112.75 ± 5.5	100.25 ± 3.96	102.82 ± 4.24	13.82 ± 0.17	7.81 ± 0.24
600	2.98 ± 0.16	121.8 ± 4.93	99.88 ± 4.12	104.39 ± 3.5	12.21 ± 0.5	8.2 ± 0.27
**Ba (µM)**	**S/R**	**TI %**			**WC (mL·g^−1^ DW)**	
		**Roots**	**Shoots**	**Entire plant**	**Roots**	**Shoots**
0	3.63 ± 0.2				13.85 ± 0.24	7.31 ± 0.14
100	2.92 ± 0.11 *	89.86 ± 6.59	75.53 ± 2.56	76.33 ± 2.27	13.1 ± 0.61	7.84 ± 0.3
200	2.85 ± 0.06 *	88 ± 4.83	70.92 ± 3.38	74.17 ± 3.43	15.73 ± 0.83	8.35 ± 0.41
400	2.94 ± 0.11 *	87.48 ± 3.09	72.57 ± 2.25	75.63 ± 1.84	15.37 ± 0.95	8.79 ± 0.28
600	2.73 ± 0.1 *	88.4 ± 2.74	67.35 ± 2.91	71.67 ± 2.51	15.39 ± 0.82	10.33 ± 0.7 *

**Table 2:** Variation of the fructification yield percentage in *Abelmoschus esculentus* L. plants subjected to increasing doses of Al and Ba (0, 100, 200, 400 and 600 µM).

Treatment (µM)	Fructification Yield (%)	
	Al	Ba
0	100%	100%
100	80%	50%
200	100%	60%
400	100%	70%
600	90%	60%

**Table 3:** Impact of the increasing doses (0, 100, 200, 400 and 600 µM) of Al and Ba on the translocation factor (TF) in *Abelmoschus esculentus* L. plants. Values are mean ± SD (*n* = 10).

Treatment (µM)	Translocation Factor (TF)	
	Al	Ba
100	0.873 ± 0.0577	0.611 ± 0.034
200	0.854 ± 0.063	0.812 ± 0.087
400	0.708 ± 0.050	0.668 ± 0.038
600	0.653 ± 0.032	0.845 ± 0.035

**Table 4:** Endogenous contents of potassium (K), calcium (Ca), magnesium (Mg), zinc (Zn) and iron (Fe) in *Abelmoschus esculentus* L. plants subjected to increasing doses of Al and Ba (0, 100, 200, 400 and 600 µM). Values are mean ± SD (*n* = 10); (*) significantly different at *p* ≤ 0.05.

**K (mg·g^−1^ DW)**	**Al (µM)**				**Ba (µM)**			
		**Roots**	**Shoots**	**Fruits**		**Roots**	**Shoots**	**Fruits**
	0	17.77 ± 0.81	23.81 ± 0.73	22.19 ± 0.42	0	17.77 ± 0.81	23.81 ± 0.73	22.19 ± 0.42
	100	11.75 ± 0.37 *	18.86 ± 0.74 *	23.75 ± 0.37	100	14.11 ± 0.67 *	20.74 ± 0.88	22.99 ± 0.53
	200	13.04 ± 0.31 *	18.90 ± 0.53 *	23.96 ± 0.52	200	14.39 ± 0.77 *	17.46 ± 1.11 *	23.02 ± 0.26
	400	13.94 ± 0.24 *	19.83 ± 0.87 *	23.26 ± 0.65	400	12.35 ± 0.29 *	17.12 ± 0.43 *	23 ± 0.5
	600	11.29 ± 0.42 *	16.41 ± 0.44 *	22.69 ± 0.7	600	13.33 ± 0.49 *	17.22 ± 0.26 *	23.73 ± 0.7
**Ca (mg·g^−1^ DW)**	**Al (µM)**				**Ba (µM)**			
		**Roots**	**Shoots**	**Fruits**		**Roots**	**Shoots**	**Fruits**
	0	8.35 ± 0.42	12.49 ± 0.41	4.71 ± 0.15	0	8.35 ± 0.42	12.49 ± 0.41	4.71 ± 0.15
	100	8.84 ± 0.55	12.44 ± 0.3	4.74 ± 0.24	100	6.85 ± 0.47	12.94 ± 0.86	3.99 ± 0.16
	200	7.01 ± 0.2 *	10.45 ± 0.26 *	4.24 ± 0.08	200	6.71 ± 0.16	10.61 ± 0.18 *	3.62 ± 0.31
	400	6.49 ± 0.47 *	9.94 ± 0.46 *	5.20 ± 0.38	400	6.82 ± 0.24	10.21 ± 0.55 *	3.78 ± 0.11
	600	6.54 ± 0.25 *	9.70 ± 0.32 *	4.37 ± 0.22	600	7.04 ± 0.24	10.94 ± 0.24 *	3.36 ± 0.16
**Mg (mg·g^−1^ DW)**	**Al (µM)**				**Ba (µM)**			
		**Roots**	**Shoots**	**Fruits**		**Roots**	**Shoots**	**Fruits**
	0	9.2 ± 0.25	6.96 ± 0.18	4.91 ± 0.11	0	9.2 ± 0.25	6.96 ± 0.18	4.91 ± 0.11
	100	8.92 ± 0.25	5.82 ± 0.14 *	4.98 ± 0.15	100	9.46 ± 0.33	7.4 ± 0.55	4.69 ± 0.2
	200	7.56 ± 0.19 *	5.5 ± 0.12 *	5.37 ± 0.23	200	10.3 ± 0.66	7.16 ± 0.32	4.85 ± 0.43
	400	7.38 ± 0.34 *	5.89 ± 0.2 *	5.61 ± 0.28	400	10.28 ± 0.32	7.01 ± 0.29	4.56 ± 0.13
	600	8.82 ± 0.19	5.43 ± 0.11 *	5.02 ± 0.12	600	10.33 ± 0.41	7.64 ± 0.39	5.47 ± 0.22
**Zn (mg·g^−1^ DW)**	**Al (µM)**				**Ba (µM)**			
		**Roots**	**Shoots**	**Fruits**		**Roots**	**Shoots**	**Fruits**
	0	0.24 ± 0.02	0.39 ± 0.02	0.13 ± 0.01	0	0.24 ± 0.02	0.39 ± 0.02	0.13 ± 0.01
	100	0.21 ± 0.01	0.21 ± 0.02 *	0.13 ± 0.01	100	0.22 ± 0.01	0.18 ± 0.02 *	0.15 ± 0.01
	200	0.15 ± 0.01 *	0.18 ± 0.03 *	0.11 ± 0.01	200	0.13 ± 0.01 *	0.09 ± 0.01 *	0.11 ± 0.02
	400	0.14 ± 0.01 *	0.13 ± 0.01 *	0.14 ± 0.02	400	0.14 ± 0.01 *	0.14 ± 0.02 *	0.14 ± 0.01
	600	0.17 ± 0.01 *	0.14 ± 0.01 *	0.14 ± 0.01	600	0.15 ± 0.01 *	0.14 ± 0.01 *	0.13 ± 0.02
**Fe (mg·g^−1^ DW)**	**Al (µM)**				**Ba (µM)**			
		**Roots**	**Shoots**	**Fruits**		**Roots**	**Shoots**	**Fruits**
	0	0.27 ± 0.01	0.22 ± 0.01	0.19 ± 0.02	0	0.27 ± 0.01	0.22 ± 0.01	0.19 ± 0.02
	100	0.26 ± 0.01	0.21 ± 0.02	0.19 ± 0.01	100	0.13 ± 0.01 *	0.18 ± 0.03	0.11 ± 0.01 *
	200	0.17 ± 0.01 *	0.14 ± 0.02 *	0.17 ± 0.01	200	0.07 ± 0.007 *	0.07 ± 0.01 *	0.09 ± 0.01 *
	400	0.18 ± 0.01 *	0.12 ± 0.01 *	0.17 ± 0.01	400	0.10 ± 0.005 *	0.03 ± 0.01 *	0.07 ± 0.003 *
	600	0.18 ± 0.01 *	0.12 ± 0.002 *	0.14 ± 0.01 *	600	0.02 ± 0.004 *	0.02 ± 0.005 *	0.09 ± 0.01 *

**Table 5:** Variation of proline, total phenols and flavonoids contents in *Abelmoschus esculentus* L. plants subjected to increasing doses of Al and Ba (0, 100, 200, 400 and 600 µM). Values are mean ± SD (*n* = 10); (*) significantly different at *p* ≤ 0.05.

**Proline**	**(µM)**	**Al**			**Ba**		
		**Roots**	**Shoots**	**Fruits**	**Roots**	**Shoots**	**Fruits**
	0	2.11 ± 0.03	1.87 ± 0.1	2.98 ± 0.09	2.11 ± 0.03	1.87 ± 0.1	2.98 ± 0.09
	100	2.19 ± 0.03	1.86 ± 0.07	2.88 ± 0.08	1.38 ± 0.08 *	2.48 ± 0.09	2.8 ± 0.08
	200	2.16 ± 0.09	2.56 ± 0.07	3.3 ± 0.17	1.48 ± 0.09 *	2.89 ± 0.09 *	3.3 ± 0.15
	400	2.58 ± 0.1 *	2.64 ± 0.03 *	3.34 ± 0.13	2.7 ± 0.12 *	2.86 ± 0.06 *	3.3 ± 0.1
	600	2.59 ± 0.08 *	2.99 ± 0.13 *	3.86 ± 0.03 *	2.71 ± 0.08 *	2.72 ± 0.24 *	3.96 ± 0.05 *
**Total phenols**	**(µM)**	**Al**			**Ba**		
		**Roots**	**Shoots**	**Fruits**	**Roots**	**Shoots**	**Fruits**
	0	10.42 ± 0.58	8.74 ± 0.70	15.25 ± 0.72	10.42 ± 1.08	8.74 ± 0.7	15.25 ± 0.72
	100	12.84 ± 0.85	17.52 ± 0.82 *	16.64 ± 1.32	10.25 ± 0.71	11.12 ± 0.5	15 ± 0.78
	200	12.35 ± 0.66	17.88 ± 0.38 *	14.56 ± 0.89	13.18 ± 0.77 *	10.61 ± 0.95	14.34 ± 1.6
	400	14.25 ± 0.44 *	20.99 ± 1.03 *	15.68 ± 0.81	13.46 ± 0.47 *	13.41 ± 0.5 *	16.02 ± 0.57
	600	17.61 ± 0.58 *	21.60 ± 2.31 *	15.87 ± 1.31	14.11 ± 0.68 *	14.05 ± 0.84 *	15.35 ± 0.8
**Flavonoids**	**(µM)**	**Al**			**Ba**		
		**Roots**	**Shoots**	**Fruits**	**Roots**	**Shoots**	**Fruits**
	0	3.89 ± 0.51	9.08 ± 0.4	6.28 ± 0.27	3.89 ± 0.51	9.08 ± 0.4	6.28 ± 0.27
	100	3.19 ± 0.31	7.48 ± 0.76	6.67 ± 0.48	1.60 ± 0.13 *	6 ± 0.52	6.43 ± 0.36
	200	2.62 ± 0.57	7.61 ± 0.2	6.20 ± 0.22	0.46 ± 0.04 *	3.34 ± 0.29 *	6.29 ± 0.15
	400	0.50 ± 0.18 *	5.64 ± 0.4	6.78 ± 0.34	0.47 ± 0.02 *	3.43 ± 0.22 *	6.92 ± 0.27
	600	0.15 ± 0.0 *	4.61 ± 0.57	7.18 ± 0.31	0.58 ± 0.05 *	3.22 ± 0.25 *	7.13 ± 0.1

## Data Availability

Not applicable.
